# Development and validation of a machine learning model to predict delays in seeking medical care among patients with breast cancer in China

**DOI:** 10.1186/s12885-025-14813-6

**Published:** 2025-09-30

**Authors:** Xiao Chen, Zhiyan Cheng, Yinfeng Li, Xiaohong Wu, Qing Yang

**Affiliations:** 1 School of Medicine, University of Electronic Science and Technology of China, Chengdu, China; 2 Kunming Medical University Haiyuan College, Kunming, China; 3 Nursing Department, Sichuan Clinical Research Center for Cancer, Sichuan Cancer Hospital and Institute, Sichuan Cancer Center, Affiliated Cancer Hospital of the University of Electronic Science and Technology of China, Chengdu, China

**Keywords:** Breast cancer, Risk factors, Machine learning, Delays among patients, Shapley additive explanations

## Abstract

**Background:**

Delays in seeking medical care may affect the survival rates of breast cancer patients. We aimed to explore potential risk factors for the delay in seeking medical care among breast cancer patients by constructing a highly effective machine learning (ML) prediction model.

**Methods:**

A cross-sectional methodology was utilized, and the demographic and clinical characteristics of 540 patients with breast cancer in Sichuan Cancer Hospital from July 2022 to June 2023 were collected to develop a model. Feature selection was performed using a Lasso algorithm, and six ML algorithms, including XGB, LR, RF, CNB, SVM and KNN, were applied for model construction. The k-fold cross-validation method was used for internal verification. And ROC curves, calibration curves, DCA and external validation were used for model evaluation. The SHAP method was used to interpret the model visualization.

**Results:**

A comprehensive analysis was conducted in a cohort of 540 patients diagnosed with breast cancer, of whom 212 patients (39.26%) experienced a delay. The Lasso algorithm selected eight variables that were most suitable for model construction. The RF model demonstrated superior performance compared to the other five prediction models. The AUC values in the training set ROC, validation set ROC, and external verification ROC curves were 1.00, 0.86, and 0.76, respectively in RF model. The results of the calibration curves indicated that the calibration curves of the RF models closely resembled the ideal curves. The DCA curves exhibited a net clinical benefit in comparison to treatment for or treatment for none for all models, with the exception of CNB.

**Conclusions:**

The machine learning algorithm utilized in this study effectively generated a prediction model for delays in seeking medical care for patients with breast cancer. The best RF model’s remarkable predictive power, exhibiting a good discrimination and calibration.

**Supplementary Information:**

The online version contains supplementary material available at 10.1186/s12885-025-14813-6.

## Introduction

Breast cancer, one of the most common types of cancer and the leading cause of cancer-related deaths among women, is the leading cause of cancer-related deaths worldwide, accounting for approximately 6.6% of the total number of cancer deaths worldwide [1].

Currently, the primary treatment for breast cancer involves surgical intervention, complemented by multimodal therapies such as radiotherapy, chemotherapy, targeted therapy, and endocrine therapy. However, breast cancer treatment is characterized by lengthy cycles, high medical expenses, and adverse effects, including the psychological impact of surgery on women’s body image and the side effects of radiotherapy and chemotherapy [2]. These multifactorial determinants collectively impair both patients’ physical and psychological health status as well as their overall quality of life, while simultaneously creating significant economic burdens for affected individuals and healthcare infrastructures. Given these substantial impacts, prompt case identification through early detection, precise diagnostic evaluation, and immediate therapeutic intervention constitute essential components for optimizing clinical outcomes. However, multidimensional barriers encompassing patient-specific factors, healthcare provider limitations, and systemic constraints frequently contribute to delayed healthcare-seeking behaviors among affected individuals. In this study, delays in seeking medical care was defined as an interval between the discovery of the initial symptoms and diagnosis, which was longer than three months [3].

Patta M et al. [4] reported that the incidence of delay in seeking medical care for patients with breast cancer was as high as 70.6%. However, delay in seeking medical care not only substantially affected the survival time but also led to an increase in medical costs, causing a serious economic burden to the country [5]. Study have demonstrated that 1.6-fold increased odds of breast cancer mortality was observed among women who experienced delay compared with women without delay4 [6]. Therefore, reducing delays in seeking medical care for breast cancer patients emerges as a crucial component of contemporary medical health care. Providing a reliable evaluation tool for delays in seeking medical care of breast cancer is critical.

Recently, some researchers have adopted traditional logistic regression to construct a prediction model for delays in seeking medical care of breast cancer. Emerging machine learning (ML) approaches have demonstrated superior predictive performance through enhanced accuracy and robust modeling capabilities [7–9]. The literature review reported that 50.2–52% of breast cancer patients in the Chinese population had a total delay interval greater than 3 months [5]. The incidence of delays in seeking medical care of breast cancer in the Chinese population was as high as 35.7% [10]. However, no studies have yet developed an ML-based predictive model specifically for Chinese breast cancer patients.

Therefore, this study systematically investigated the influencing factors of delay in seeking medical care among breast cancer patients from the aspects of sociodemographic, environmental, and clinical variables. and developed a predictive machine learning model that incorporated the independent effects and interactions of these factors to assess the risk of delayed medical consultations for breast cancer patients in the Chinese population. This model can be utilized by individual physicians and patients to evaluate the risk and make targeted decisions regarding delayed breast cancer screening. Simultaneously, health care systems can employ this model to identify high-risk patients, enabling the implementation of additional interventions at the population level.

## Materials and methods

### Study design

A cross-sectional methodology was utilized in this study. This study was approved by the Institutional Review Board of Sichuan Cancer Hospital (approval number: SCCHEC-02-2022-086). All participants signed informed consent forms and were asked to complete the structured questionnaires independently after giving their consent.

### Patients

A total of 540 patients with breast cancer in Sichuan Cancer Hospital in China from July 2022 to June 2023 were recruited for model construction. Additionally, 150 breast cancer patients from the same hospital during a different time period (July to August 2023) were selected as the study subjects for external validation of the model. The inclusion criteria included (a) aged over 18 years; (b) having pathologically diagnosed breast cancer; (c) having normal linguistic and comprehension abilities; and (d) consenting to participate in this study. The exclusion criteria were as follows: (a) a history of mental illness or cognitive disorders; (b) other comorbid cancers; and (c) relapsed breast cancer.

### Data collection

We collected 35 independent variables, encompassing sociodemographic, environmental and clinical factors.

#### Sociodemographic characteristics

 We designed a sociodemographic characteristics questionnaire based on a literature review to collect the participants’ sociodemographic information, including age, alcohol consumption, smoking status, ethnicity, religion, marital status, employment, family history of any cancer, family history of breast cancer, number of family members, hospital choice, hospital that confirmed breast cancer, place of residence, education level, monthly income (RMB), distance from the hospital, household situation, channels for acquiring health knowledge, preferred solution for breast discomfort, barriers and reasons for delaying seeking medical care, health care worker status, physical examination status, understanding of breast diseases, comorbidities, and medical payment method.

#### Clinical characteristics

 Additionally, we designed a clinical characteristics questionnaire reflecting medical treatment-seeking behavior, including clinical stage, first detected symptoms, method of symptom discovery, when the symptoms were first discovered, and when the patient sought medical treatment for the first time.

#### Anxiety

 Anxiety was evaluated using the Generalized Anxiety Disorder Scale (GADS-7), which consisted of 7 items [11]. The Cronbach’s alpha coefficient is 0.907. Responses to this self-report scale range from 0 (completely absent) to 3 (almost every day). The scores range from 0 to 21, with higher scores indicating higher levels of anxiety.

#### Depression

 Depression was assessed using the Patient Health Questionnaire Depression Scale (PHQ-9), which consisted of 9 items [12]. The Cronbach’s alpha coefficient is 0.767. Responses to this self-report scale range from 0 (almost none) to 3 (every day). The scores range from 0 to 27, with higher scores indicating higher levels of depression.

#### Family support

 Family support was evaluated using the Family Support Scale (FSS), which consisted of 15 items [13]. The Cronbach’s alpha coefficient is 0.83. The FSS contains 15 items, forming a three-level scoring system, from 3= “fully compliant” to 1= “completely noncompliant”. The scores range from 15 to 45, with higher scores indicating higher levels of family support.

#### Medical coping mode

 The medical coping mode was assessed using the Medical Coping Mode Questionnaire (MCMQ), which consisted of 20 items across three dimensions: confrontation, avoidance, and acceptance resignation [14]. The Cronbach’s alpha for each dimension ranges from 0.69 to 0.76. Responses to this self-report scale are measured using a 4-point Likert scale, with scores ranging from 1 to 4.

#### Health hardiness

The RHHI-24 was developed based on the Health-Related Hardiness Scale (HRHS), and consisted of 24 items and four dimensions, including health values(reflect the study subjects’ level of concern regarding their health status), internal health locus of control, external health locus of control, and perceived health competence [15]. The Cronbach’s alpha for each dimension ranges from 0.66 to 0.79. Responses to this self-report scale are measured using a 5-point Likert scale, with scores ranging from 1 (completely disagree) to 5 (completely agree).

### Outcome

The primary outcome variable was delay in seeking medical care, which was defined as a delay in diagnosis (referred to as patient delay) if the duration between the patient’s initial observation of suspicious symptoms and their first visit to a medical institution exceeds 3 months [3].

### Statistic analysis

#### Establishment of predictive models

In the present study, all statistical analyses were carried out using R version 3.6 and Python version 3.7. Continuous variables were described as means [standard deviations (SDs)] or medians [25th, 75th percentiles], while categorical variables were expressed as numbers (percentages). T tests, Mann‒Whitney U tests, and Chi-square tests were used to identify variables displaying significant differences between the non-delayed group and the delayed group. Additionally, a least absolute shrinkage and selection operator (LASSO) analysis was conducted for dimension reduction to identify the most appropriate predictors for building the machine learning model [16]. The individuals included in the study were randomly assigned to a training set and a validation set at a ratio of 7:3, with 378 participants in the training set and 162 participants in the validation set. Six machine learning methods, including extreme gradient boosting (XGB), logistic regression (LR), random forest (RF), complementary naive bayes (CNB), support vector machine (SVM) and k-nearest neighbor (KNN), were applied for model construction were used for model construction.

#### Evaluation of predictive models

 The six models were evaluated based on three aspects: discrimination, calibration, and clinical usefulness, with the best model selected for predictive analysis. The receiver operating characteristic (ROC) curve was plotted to obtain the area under the curve (AUC) value, which reflects the model’s predictive efficiency [17]. A calibration curve was created to assess the model’s performance, using calibration plots to evaluate the deviation of model predictions from actual events [16]. Decision curve analysis (DCA) was conducted to calculate the net benefits at various threshold probabilities [18]. The intersection of the red curve and the treat all curve was the starting point, and the intersection of the red curve and the treat none curve was the node, within which the corresponding patients could benefit. The k-fold cross-validation method was employed for internal validation. For robust model parameter optimization, we systematically implemented 10-fold cross-validation as the internal validation strategy. The standardized protocol involved: (1) random partitioning of the complete dataset into 10 equally sized subsets, (2) iterative training on 9 subsets with validation on the remaining subset, and (3) repetition of this process across all possible combinations. This rigorous approach generated stable parameter estimates while maintaining maximal data utility. The final model architecture incorporated L2 penalty regularization to effectively prevent overfitting while preserving predictive performance. The best model was externally validated using an external test set, the ROC curve was plotted, and the generality and predictive efficacy of the model were determined.

#### Model interpretation

 The shapley additive explanations (SHAP) method was used to interpret the model visualization to measure the importance of eigenvalues, ranking the importance of each predictor according to its corresponding SHAP value [19].

## Results

### Patient characteristics

The comparison of the demographic and clinical characteristics observed in the training set and the verification set was provided in supplementary 1.

### Predictors variables selection based on Lasso

**Fig. 1:**
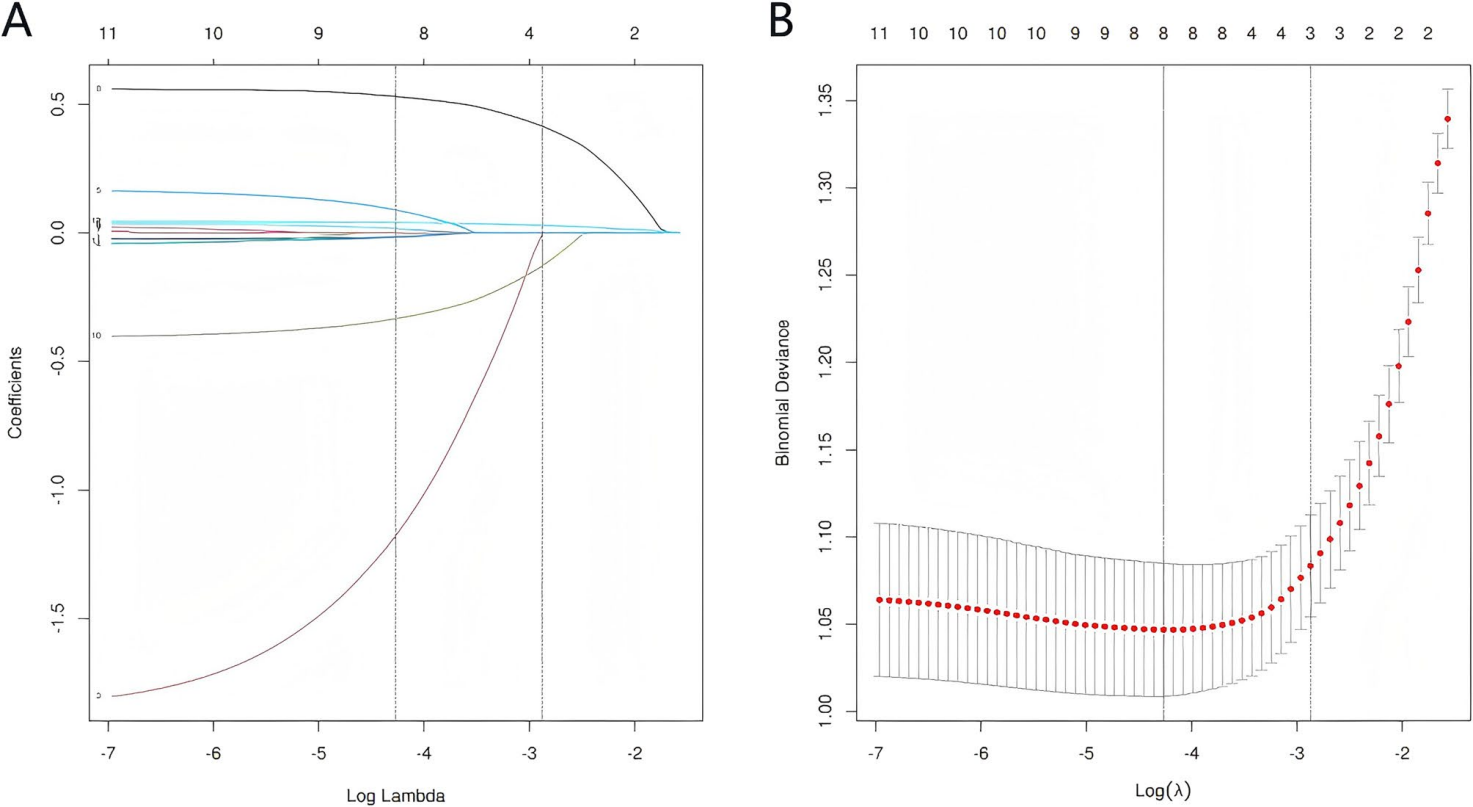
Variable selection was performed using the LASSO elastic net approach.(A) Coefficient profile plot.(B) Cross-validation error plot

We employed the Lasso algorithm to identify features relevant for classifying the 12 variables that exhibited significant differences (Table 1). The results of feature screening based on the Lasso algorithm are illustrated in Fig. 1 A, B. As log (λ) increases, the average standard error also increases, leading to varying degrees of compression in the normalization coefficients of the 12 candidate variables until all coefficients reach zero. X-axis (Lambda): Represents the value of regularization parameter λ. As λ increases, the model complexity decreases, with more coefficients being shrunk to zero. Y-axis: Displays the coefficient values of each variable. Curves: There are 12 colored lines corresponding to the 12 included variables, each labeled with a variable number at its end. Each curve represents the coefficient path of an individual predictor. Ultimately, we identified eight predictive variables selected for developing the machine learning models, including health value, distance from the hospital, religion, education level, physical examination status, medical choice, preferred solutions for breast discomfort, and medical payment method.

**Table 1:** Demographic and clinical characteristics of the participants

Variables	Non-delay (*n* = 328)	Delay (*n* = 212)	Total(= 540	Statistic			*P*
Place of residence, n (%)							
Rural	146(44.5%)	109(51.4%)	255(47.22%)		2.46	0.117	
Urban	182(55.5%)	103(48.6%)	285(52.78%)				
House hold situation, n (%)							
Alone	12(3.7%)	13(6.1%)	25(4.63%)		1.78	0.182	
Social	316(96.3%)	199(93.9%)	515(95.37%)				
Number of family members, n (%)							
One-three	136(41.5%)	91(42.9%)	227(42.04%)		0.42	0.812	
Four-five	145(44.2%)	88(41.5%)	233(43.15%)				
Six and above	47(14.3%)	33(15.6%)	80(14.81%)				
Ethnicity, n (%)							
Han Chinese	324(98.8%)	203(95.8%)	527(97.59%)		5.02	0.025	
Others ethnicity	4(1.2%)	9(4.2%)	13(2.41%)				
Religion, n (%)							
Yes	3(0.9%)	13(6.1%)	16(2.96%)		12.19	< 0.001	
No	325(99.1%)	199(93.9%)	524(97.04%)				
Education levels, n (%)							
Illiterate	19(5.8%)	26(12.3%)	45(8.33%)		15.66	0.008	
Primary school	76(23.2%)	53(25.1%)	129(23.89%)				
Middle school	100(30.5%)	77(36.3%)	177(32.78%)				
High school	54(16.5%)	23(10.8%)	77(14.26%)				
Junior college	43(13.1%)	20(9.4%)	63(11.67%)				
University or above	36(10.9%)	13(6.1%)	49(9.07%)				
Employment, n (%)							
Farmer	139(42.4%)	101(47.6%)	240(44.44%)		11.7	0.111	
Worker	22(6.7%)	20(9.4%)	42(7.78%)				
Businessmen	13(4%)	6(2.8%)	19(3.52%)				
Service industry	33(10%)	16(7.5%)	49(9.07%)				
Management industry	17(5.2%)	6(2.8%)	23(4.26%)				
Technical industry	16(4.9%)	2(0.9%)	18(3.33%)				
Retirement	41(12.5%)	25(11.8%)	66(12.22%)				
Others	47(14.3%)	36(16.9%)	83(15.37%)				
Marital status, n (%)							
Married	294(89.6%)	184(86.8%)	478(88.52%)		2.51	0.473	
Unmarried	6(1.8%)	6(2.8%)	12(2.22%)				
Divorced	16(4.9%)	9(4.2%)	25(4.63%)				
Widowed	12(3.7%)	13(6.2%)	25(4.63%)				
Monthly income (RMB), n (%)							
Below 3000	145(44.2%)	117(55.2%)	262(48.52%)		9.23	0.026	
3000–5000	123(37.5%)	70(33%)	193(35.74%)				
5000–8000	33(10.1%)	18(8.5%)	51(9.44%)				
More than 8000	27(8.2%)	7(3.3%)	34(6.3%)				
Smoking status, n (%)							
Never	308(94%)	198(93.4%)	506(93.7%)		3.47	0.185	
Occasionally	8(2.4%)	10(4.7%)	18(3.33%)				
Often	12(3.6%)	4(1.9%)	16(2.96%)				
Alcohol status, n (%)							
Never	245(74.7%)	167(78.8%)	412(76.3%)		3.12	0.21	
Occasionally	74(22.6%)	36(17%)	110(20.37%)				
Often	9(2.7%)	9(4.2%)	18(3.33%)				
Medical payment method, n (%)							
Self-payment	19(5.8%)	11(5.2%)	30(5.56%)		9.74	0.008	
Employee medical insurance	116(35.4%)	49(23.1%)	165(30.56%)				
New rural health insurance	193(58.8%)	152(71.7%)	345(63.89%)				
Comorbidities, n (%)							
No	254(77,4%)	161(75.9%)	415(76.85%)		0.16	0.687	
Yes	74(22.6%)	51(24.1%)	125(23.15%)				
Conscious severity of breast disease, n (%)							
Not serious	48(14.6%)	30(14.1%)	78(14.44%)		2.32	0.509	
Moderate	111(33.8%)	60(28.3%)	171(31.67%)				
Rather serious	127(38,7%)	89(42%)	216(40%)				
Very serious	42(12.9%)	33(15.6%)	75(13.89%)				
Status of understanding breast diseases, n (%)							
Understand	99(30.2%)	77(36.3%)	176(32.59%)		2.21	0.137	
Don’t understand	229(69.8%)	135(63.7%)	364(67.41%)				
Physical examination status, n (%)							
Once a year	90(27.4%)	48(22.6%)	138(25.56%)		37.79	< 0.001	
Every two years	34(10.4%)	10(4.7%)	44(8.15%)				
Three years or above	18(54.9%)	3(1.4%)	21(3.89%)				
Occasionally	103(31.4%)	46(21.7%)	149(27.59%)				
Never	83(25.3%)	105(49.5%)	188(34.81%)				
Healthcare workers, n (%)							
No	265(80.8%)	188(88.7%)	453(83.89%)		5.93	0.015	
Yes	63(19.2%)	24(11.3%)	87(16.11%)				
Medical choice, n (%)							
First level hospital	228(69.5%)	69(32.5%)	297(55%)		77.45	< 0.001	
Second level hospital	32(9.8%)	26(12.3%)	58(10.74%)				
Third level hospital	63(19.2%)	107(50.5%)	170(31.48%)				
The small clinic	5(1.5%)	10(4.7%)	15(2.78%)				
Barriers and reasons for the delay in seeking medical care, n (%)							
Economic hardship	200(61%)	101(47.6%)	301(55.74%)		21.44	0.002	
Mild symptoms of self-awareness	69(21%)	75(35.4%)	144(26.67%)				
Unaccompanied	27(8.2%)	9(4.2%)	36(6.67%)				
Too far away	18(5%)	11(5.2%)	29(5.37%)				
Untreatable	3(0.9%)	1(0.5%)	4(0.74%)				
Taboo/avoidance	9(3%)	11(5.2%)	20(3.7%)				
No time available	2(0.9%)	4(1.9%)	6(1.11%)				
The preferred solution for feeling breast discomfort, n (%)							
Not seeking medical treatment	8(2.4%)	30(14.2%)	38(7.04%)		41.7	< 0.001	
Taking medicine	45(14%)	51(24.1%)	96(17.78%)				
Seeking medical facility	273(83%)	129(60.8%)	402(74.44%)				
Online consultation	2(0.6%)	2(0.9%)	4(0.74%)				
Channels for acquiring health knowledge, n (%)							
Not interested	50(15.2%)	52(24.5%)	102(18.89%)		12.49	0.052	
TV	70(21.3%)	36(17.1%)	106(19.63%)				
Magazine	14(4.3%)	5(2.3%)	19(3.52%)				
Online	95(29.1%)	58(27.3%)	153(28.33%)				
Health lecture	9(2.7%)	2(0.9%)	11(2.04%)				
Talking with family	87(26.5%)	54(25.5%)	141(26.11%)				
Training course	3(0.9%)	5(2.4%)	8(1.48%)				
First detected symptoms, n (%)							
Breast lump	291(88.7%)	189(89.2%)	480(88.89%)		5.8	0.214	
Nipple ulcers/itching	3(1%)	4(1.9%)	7(1.3%)				
Change in breast shape	4(1.2%)	7(3.3%)	11(2.04%)				
Nipple discharge4	6(1.8%)	3(1.4%)	9(1.67%)				
No symptom (no examination)	24(7.3%)	9(4.2%)	33(6.11%)				
Method of symptom discovery, n (%)							
Pain stimulation	52(15.8%)	45(21.2%)	97(17.96%)		2.79	0.425	
Accidental discovery	165(50.3%)	103(48.6%)	268(49.63%)				
Breast self-examination	56(17.1%)	34(16%)	90(16.67%)				
Physical/clinical breast examination	55(16.8%)	30(14.2%)	85(15.74%)				
Companion, n (%)							
Spouse	214(65.2%)	135(63.7%)	349(64.63%)		2.35	0.309	
Family	88(26.8%)	52(24.5%)	140(25.93%)				
Friends	26(8%)	25(11.8%)	51(9.44%)				
Hospitals with confirmed breast diseases, n (%)							
Third level hospital	217(66.1%)	144(68%)	361(66.85%)		0.73	0.693	
Second level hospital	57(17.4%)	31(14.6%)	88(16.3%)				
First level hospital	54(16.5%)	37(17.4%)	91(16.85%)				
Pathologic stage, n (%)							
I	103(31.4%)	66(31.1%)	169(31.3%)		6.07	0.108	
II	126(38.4%)	63(29.7%)	189(35%)				
III	51(15.5%)	40(18.9%)	91(16.85%)				
IV	48(14.7%)	43(20.3%)	91(16.85%)				
Family history of breast cancer, n (%)							
vyes	18(5.5%)	11(5.2%)	29(5.37%)		0.02	0.88	
No	310(94.5%)	201(94.8%)	511(94.63%)				
Family history of other cancer, n (%)							
Yes	23(7.1%)	24(11.3%)	47(8.7%)		3.01	0.083	
No	305(92.9%)	188(88.7%)	493(91.3%)				
Age at diagnosis years, n (%)							
18–44	83(25.3%)	39(18.4%)	122(22.59%)		3.57	0.167	
45–59	195(59.5%)	136(64.2%)	331(61.3%)				
≥ 60	50(15.2%)	37(17.4%)	87(16.11%)				
Distance from the hospital, median [IQR]	9.00[4.00,18.00]	41.00[20.00,50.00]			−11.55	< 0.001	
Anxiety, median [IQR]	4.00[0.00,7.00]	4.00[0.00,7.00]			−0.34	0.731	
Depression, median [IQR]	4.00[1.00,7.00]	4.00[1.00,8.00]			−0.45	0.651	
Family support, median [IQR]	34.00[32.00,36.00]	34.00[33.00,36.00]			−0.39	0.692	
Medical coping mode							
Confrontation, median [IQR]	21.00[19.00,22.00]	21.00[19.00,22.00]			0.78	0.43	
Avoidance, median [IQR]	17.00[15.00,19.00]	17.00[15.00,18.00]			1.52	0.126	
Acceptance resignation, median [IQR]	14.00[13.00,15.00]	14.00[13.00,15.00]			0.63	0.52	
Health hardiness							
Health value, median [IQR]	23.00[21.00,24.00]	23.00[19.00,24.00]			2.29	0.02	
Internal health locus of control, median [IQR]	18.00[15.00,20.00]	17.00[15.00,19.00]			1.81	0.069	
External health locus of control, median [IQR]	20.00[17.00,23.00]	20.00[17.00,23.00]			−0.19	0.847	
Perceived health competence, median [IQR]	20.00[17.00,22.00]	19.00[17.00,21.00]			1.04	0.296	

### Model building and evaluation

#### Establishment of predictive models

**Fig. 2:**
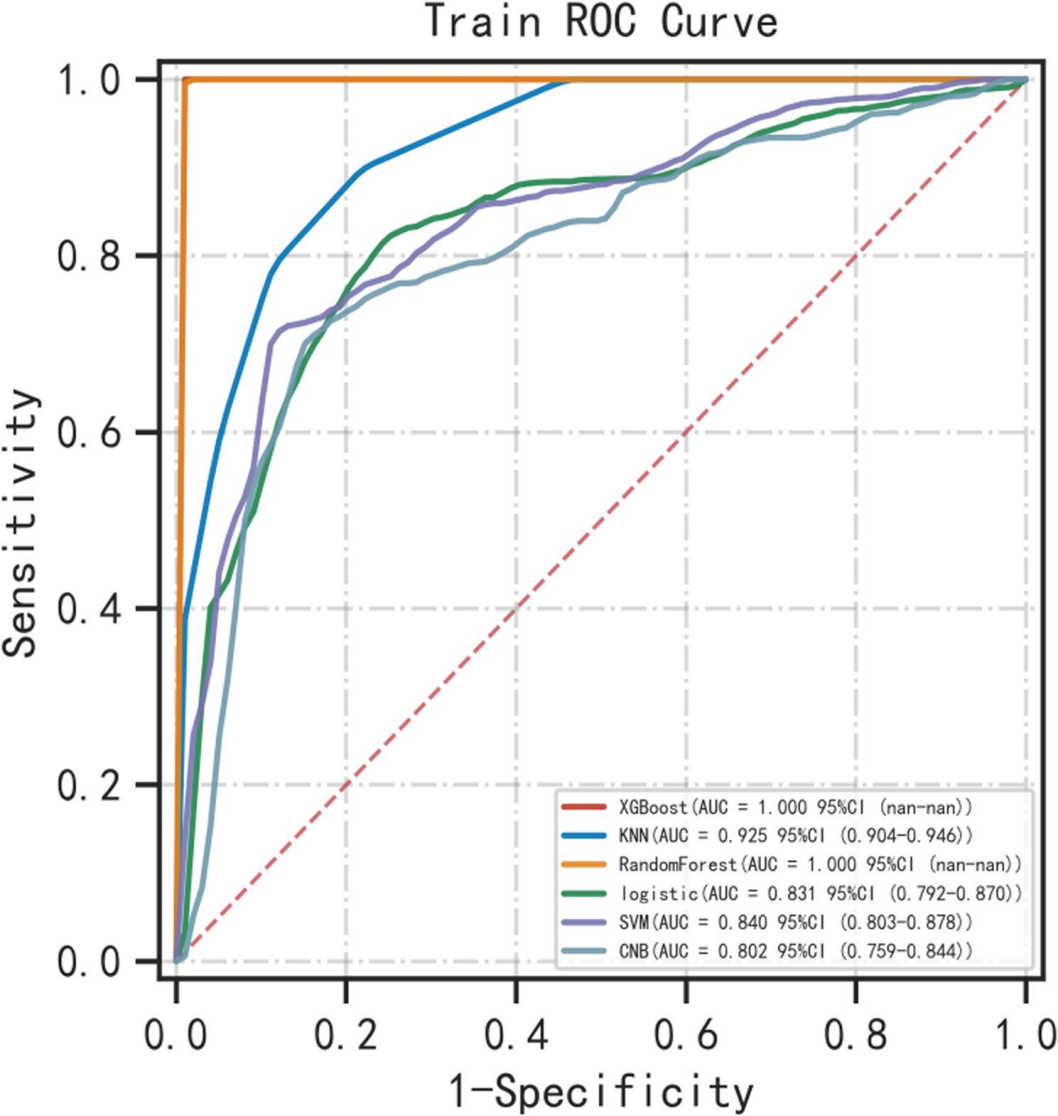
ROC curves for the training set of the six models

**Fig. 3:**
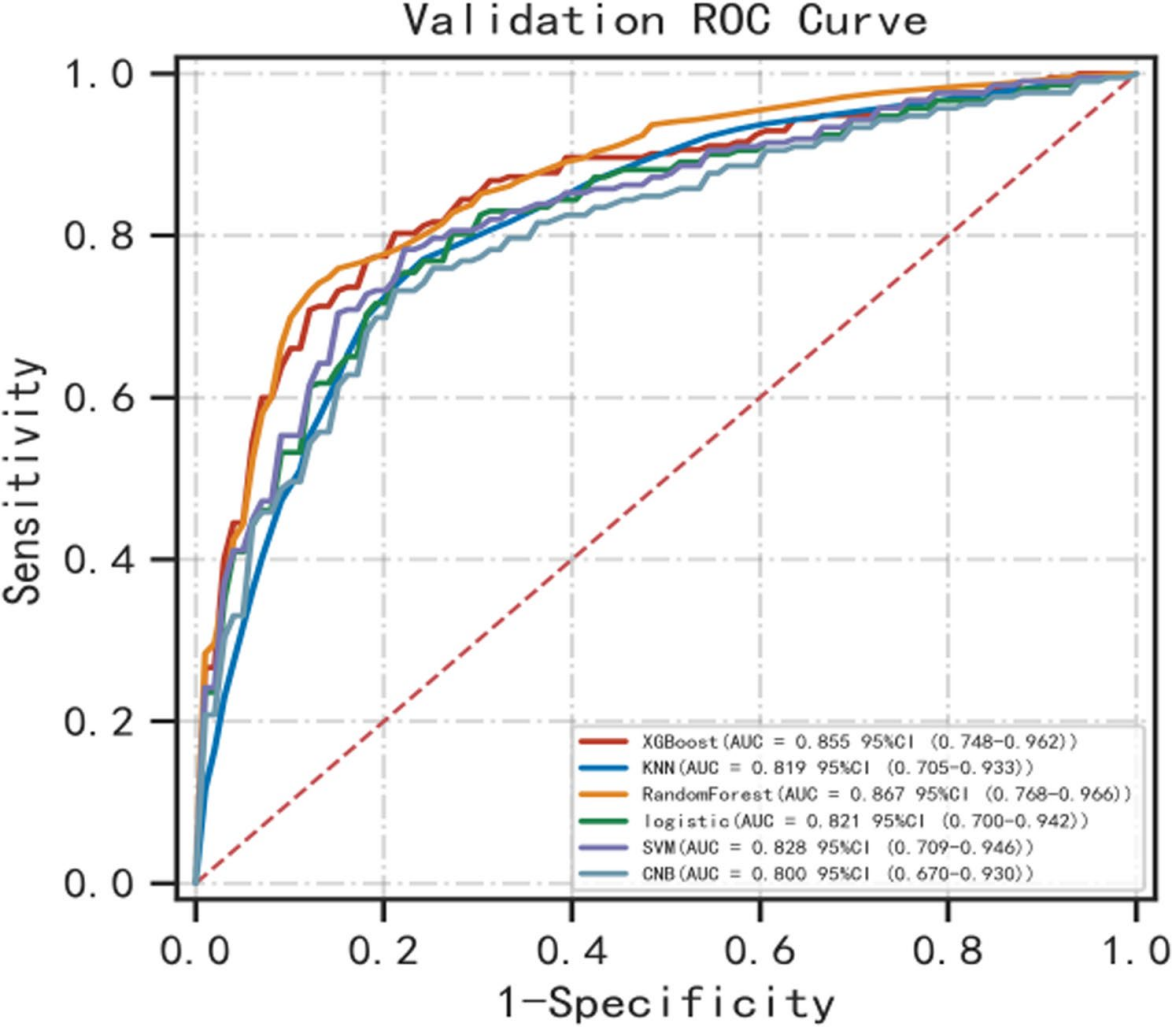
ROC curves for the validation set of the six models

**Fig. 4:**
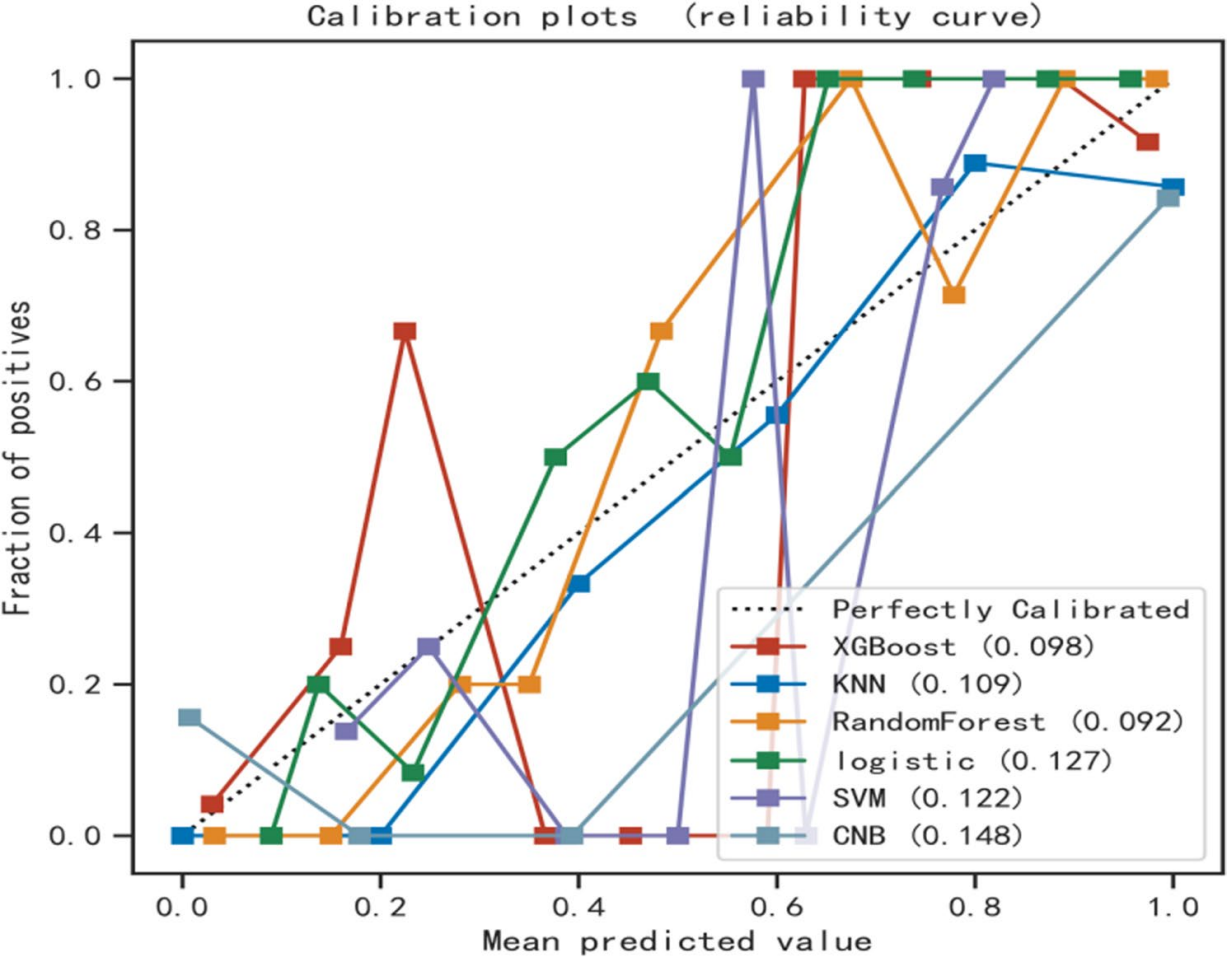
Calibration plots of the six models

**Fig. 5:**
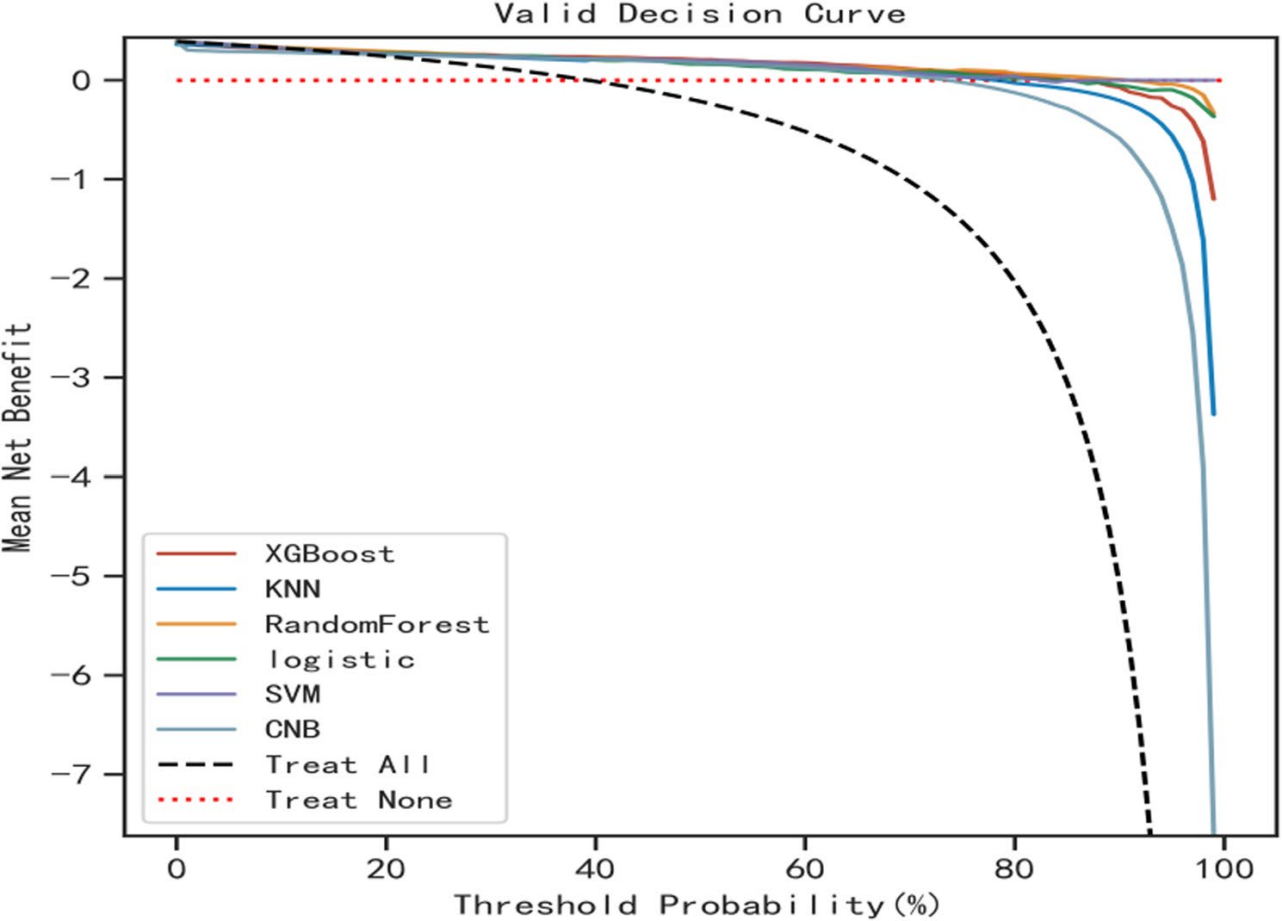
DCA curves of the six models

**Fig. 6:**
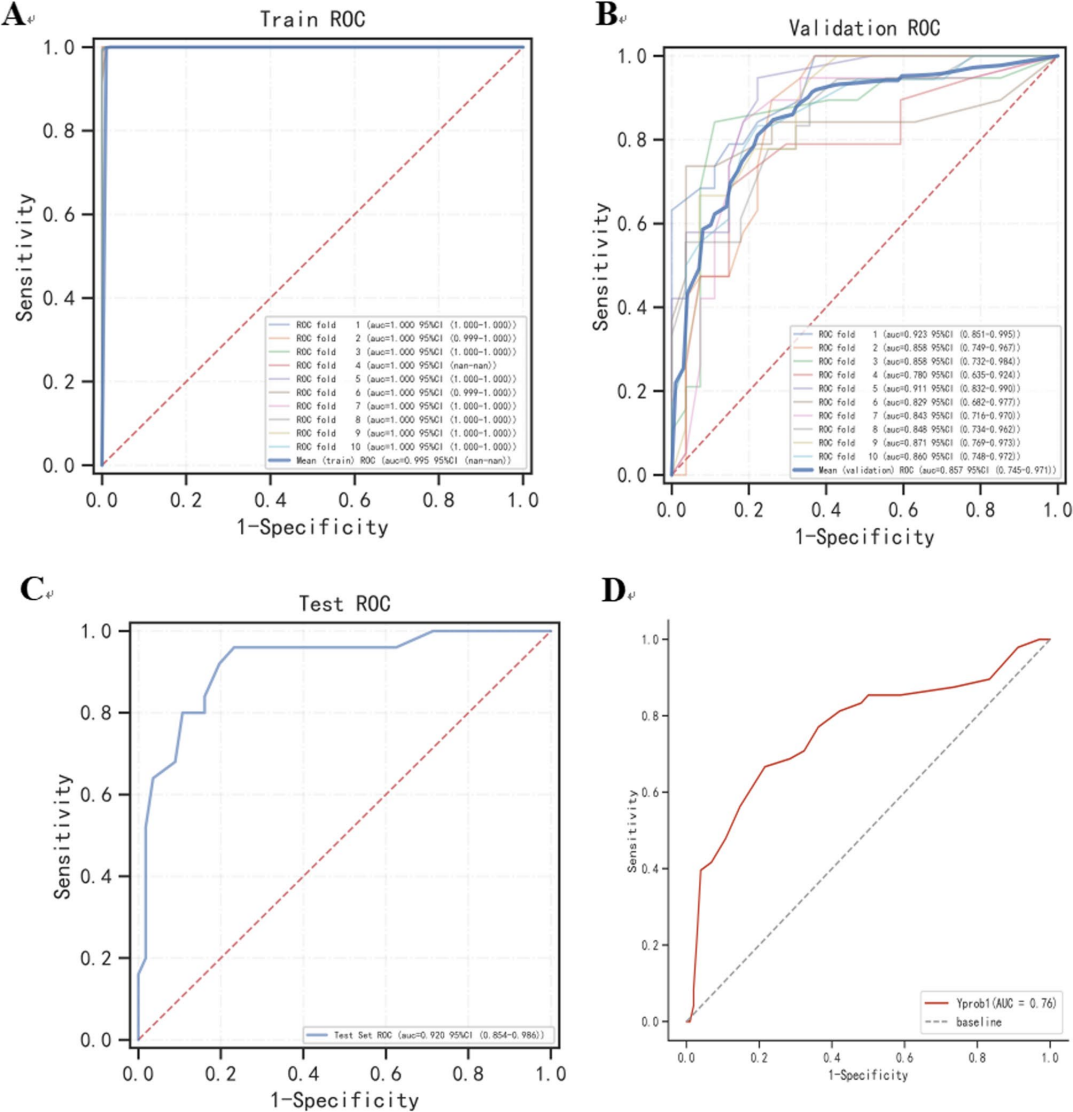
**A** ROC curve of the RF model for the training set.(**B**) ROC curve of the RF model for the validation set.(**C**) ROC curve of the RF model for the test set.(**D**) External validation of the RF model

Six machine learning algorithms were employed to build a prediction model in this study. The training set was used to create and train the machine learning models. After adjusting for parameters and comparing algorithms, the extreme gradient boosting (XGB), logistic regression (LR), random forest (RF), CNB, support vector machine (SVM) and k-nearest neighbor (KNN) models were established, and the average AUC values were greater than 0.80(Table 2). The experimental results showed that RF was the optimal model, And the AUC values of the training set And validation set were 1.00 And 0.87, respectively, both of which performed the best among the sets of the six models (Figs. 2 and 3). The results of the calibration curves indicated that the calibration curves of the RF models closely resembled the ideal curves, and there was a significant agreement between the predicted and actual outcomes of these models (Fig. 4). The DCA curves exhibited a net clinical benefit in comparison to treatment for all or treatment for none models, with the exception of CNB (Fig. 5). The k-fold cross-validation method was applied to the RF models, the test set of 38 cases (10%) was taken, and the remaining samples were used as the training set for 10-fold cross-validation. The results showed a mean (training) AUC = 0.955, mean (validation) AUC = 0.857, and mean (test) AUC = 0.920 for the RF models (Fig. 6A, B, C). The prediction model was built using RF after the combined comparison.

**Table 2:** Evaluation of the six model

Model	Training				Validation			
	AUC	ACC	SN	SP	AUC	ACC	SN	SP
XGBoost	1.00	1.00	1.00	1.00	0.85	0.82	0.82	0.84
KNN	0.92	0.82	0.84	0.85	0.82	0.77	0.75	0.81
RF	1.00	0.99	1.00	0.99	0.87	0.79	0.77	0.86
LR	0.83	0.78	0.82	0.77	0.82	0.77	0.82	0.78
SVM	0.84	0.82	0.71	0.89	0.83	0.81	0.76	0.87
CNB	0.80	0.79	0.71	0.85	0.80	0.79	0.75	0.85

*RF* Random Forest, *XGBoost* Extreme Gradient Boosting, *LR* Logistic Regression, *SVM* Support Vector Machine, *CNB* Complementary naive Bayes, *KNN* K-nearest Neighbor, *AUC* Metrics Area Under ROC Curve, *ACC* Accuracy, *SN* Sensitivity, *SP* Specificity

### Model external validation

The ROC curve analysis yielded An AUC value of 0.76 for the external validation set, suggesting a strong alignment between the predicted and actual probabilities. Furthermore, the extrapolative nature of the predictive model was evident (Fig. 6D).

### Model explanation

**Fig. 7:**
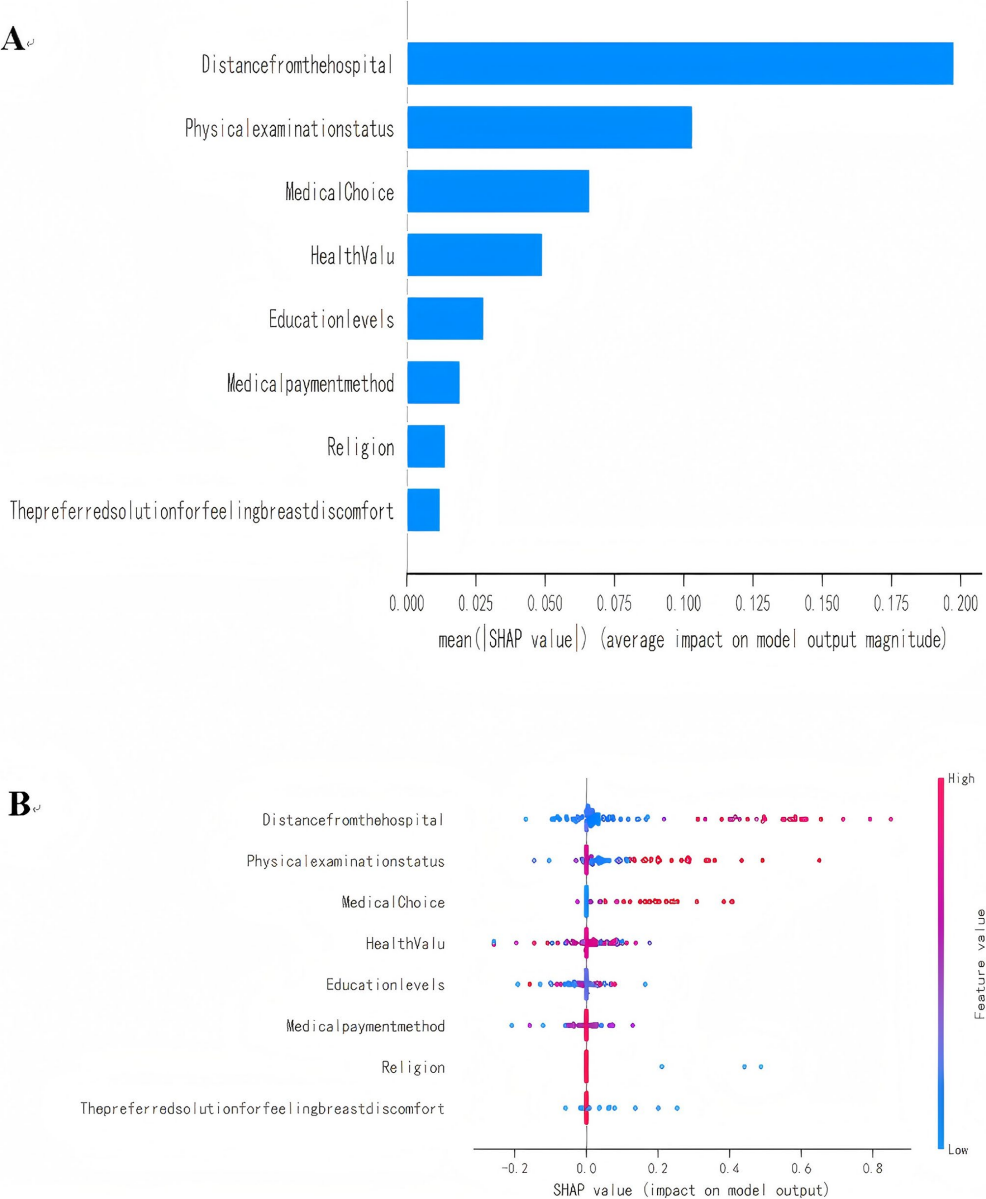
Bar plot of the feature importance. (**A**) Beeswarm plot of effects of features. **B**

The SHAP summary plot of feature importance results demonstrated the hierarchical ranking of the eight risk factors associated with delays in seeking medical attention in terms of their influence as distance from the hospital, physical examination status, hospital choice, health value, monthly income (RMB), education level, religion, and medical payment method (Fig. 7).

## Discussion

In this study, we developed and evaluated six machine learning predictive models (RF, LR, XGB, KNN, SVM, and CNB) for delays in seeking medical care among breast cancer patients. Among these models, the RF model demonstrated the highest prediction performance, with an effective AUC value (Table 2). The AUC values in the training set ROC, validation set ROC, and external verification ROC curves were 1.00, 0.87, And 0.76, respectively. To date, the investigation conducted by Samira et al. remains the sole study that has developed a machine learning-based predictive model for delayed healthcare-seeking behavior in breast cancer patients, with their research focused specifically on the Iranian population [20]. Notably, their findings identified the Random Forest (RF) algorithm as demonstrating optimal predictive performance among the models evaluated. However, several critical limitations should be acknowledged: (1) the study exclusively employed internal validation methods, and (2) comparative analysis reveals that the RF model’s performance metrics in their internal validation were substantially inferior to those achieved by our proposed model (Accuracy: 0.70 vs. 0.79; Sensitivity: 0.837 vs. 0.77; Specificity: 0.361 vs. 0.86; AUC: 0.788 vs. 0.87). This performance disparity highlights the superior discriminative capacity And clinical utility of our model. Furthermore, to ensure robust generalizability of our predictive model, we conducted comprehensive external validation utilizing An independent patient cohort, thereby demonstrating its clinical applicability across diverse populations. which yielded an AUC value of 0.76. Furthermore, the study utilized the SHAP approach to visualize the model, addressing the “black box” issue commonly associated with machine learning predictive models. The results of this study confirmed that the predictive model for delays in seeking medical care for breast cancer developed for the Chinese population has better external generalizability.

This study demonstrated that the prevalence of patient delay was 39.3%, which was similar to those reported in previous studies [8]. Breast cancer patients may be influenced by various factors that lead them to disregard their problems and postpone seeking medical care. SHAP analysis revealed the significant predictors of delays in seeking medical care among breast cancer patients, including, distance from the hospital, physical examination status, hospital choice, health value, education level, medical payment method, preferred solution for breast discomfort, and religion, these eight variables were readily available in both hospital and community settings, and involved almost no cost, which facilitated the subsequent promotion and application of the model.

The Fig. 7 showed that the distance from hospital was the most important predictor of seeking medical care among patients with breast cancer. Studies have confirmed that access to health care was an important factor in delaying medical treatment for breast cancer patients [21, 22]. And studies have also confirmed that living in rural areas was an important risk factor for delays in seeking medical care of breast cancer [23, 24]. Hence, improving the accessibility of healthcare services is crucial for reducing delays in seeking medical care for patients with breast cancer.

Our study found that physical examination status was the second most significant predictor of delays in seeking medical care among patients with breast cancer. In this study, 34.81%(188/540) of the patients had not undergone Any physical examination, and among those who had never undergone a physical examination, 55.85%(105/188) experienced delays in seeking medical care. A study noted that in the physical examination of malignant tumors in healthy people [25], the more the subjects knew about malignant tumors, the more likely they were to seek medical consultation early, and the lower the possibility of delayed medical treatment, which is consistent with the results obtained in this study.

Educational level and health values were also indicators affecting medical-seeking behavior. Other studies have indicated that education level is also associated with delayed presentation, which reported findings similar to our study [2, 24, 26]. Individuals with lower levels of education tend to exhibit prolonged delays in seeking medical care. Moreover, from the collected questionnaires, most of the people with lower education levels had lower health values. Therefore, improving education levels and enhancing the dissemination of knowledge related to breast cancer are crucial for reducing delays in seeking medical care in breast cancer.

The preferred solution for breast discomfort was also correlated with patient delay in this study, which is consistent with the findings of Ren et al. [27]. Our study has confirmed the distance from hospital was the most important factor contributing to the delay in seeking medical attention for breast cancer patients. When patients felt breast discomfort, they preferred to visit a small clinic. The reason may be that women play an important role in caring for children and families in China, and may prefer small clinics close to home to buy medicine and may only visit hospitals when serious symptoms appear. However, due to the poor diagnosis and treatment level of the clinic, some patients obtained the wrong symptom explanation, resulting in a delay.

The medical payment method was an important predicted indicator affecting seeking medical care in our study. The lack of health insurance and limited access to medical services contribute to patients’ delay in seeking necessary medical attention. Rauscher et al. [21] also found that the availability and utilization of health care services strongly predicted patient delays in seeking medical care. Patients with medical insurance and regular visits to doctors were less likely to experience delays. Nelissen et al. [22] observed that individuals tend to assess their available health care resources before seeking medical help. Factors such as the absence of medical insurance, difficulties in accessing medical services, and complex referral processes all act as barriers for patients seeking timely medical assistance.

Our study identified that religion Religious belief serves as a potential contributing factor to patient delay in seeking medical care. In this study, the proportion of breast cancer patients with religious beliefs experiencing delays in seeking medical care was as high as 81.25%, which was closely related to the research setting of this study. This study was conducted in a tertiary first-class oncology specialized hospital in Sichuan, which treated a majority of cancer patients from the Sichuan region. Sichuan had a very unique geographical location, and the majority of the population with religious beliefs was Tibetan. Tibetans mostly resided in remote plateau areas where medical resources were limited, and the overall educational level among the Tibetan people tended to be relatively low. Ma et al. [28] also identified that religious belief(fear of stigmatization) was a risk factor for delays in seeking medical care in women with cervical cancer, which was similar to the findings of our study. However, data on religious affiliation were limited, with only 16 cases (2.69%) reporting religious beliefs. Within this cohort, prompt healthcare-seeking behavior was observed in 3 cases (18.75%), whereas delayed presentation was identified in the remaining 13 patients (81.25%). Given the small sample size, future studies will expand data collection through multicenter investigations at tertiary hospitals in ethnic minority regions to enhance the representativeness of this demographic characteristic.

### Limitations

Although the model developed in this study demonstrated excellent discrimination ability, calibration, and clinical effectiveness, there are several limitations. First, the data was derived from self-reports obtained through questionnaires, which may have introduced bias. Second, this study included data from only a single center, and the sample size was small, which may have led to distribution bias and may not be ideal for validating datasets in model development. Therefore, it is essential to obtain multicenter clinical data to provide more reliable theoretical guidance for clinical practice. Additionally, it is necessary to update the prediction model and enhance its performance.

## Conclusions

The machine learning algorithm utilized in this study effectively generated a prediction model for delays in seeking medical care for patients with breast cancer. The best RF model’s remarkable predictive power, exhibiting a good discrimination and calibration, which could intuitively and easily screen those at high risk of delaying seeking medical care in the community and provide a reliable evaluation tool for early screening and intervention to reduce delays.

## Supplementary Information

Supplementary Material 1.

Supplementary Material 2.

## Data Availability

The raw data supporting the conclusions of this article will be made available by the authors without undue reservation.

## Abbreviations

XGB: Extreme gradient boosting
LR: Logistic regression
RF: Random forest
CNB: Complementary naive bayes
SVM: Support vector machine
KNN: K-nearest neighbor
GAD-7: Generalized anxiety disorder scale
PHQ-9: Patient health questionnaire depression scale
FSS: Family support scale
MCMQ: Medical coping mode questionnaire
HRHS: Health-related hardiness scale
ROC: Receiver operating characteristic
AUC: Area under the curve
DCA: Decision curve analysis
SHAP: SHapley additive explanations
LASSO: Least absolute shrinkage and selection operator

## References

[1] Li S, Wu D, Jia H, et al. Long non-coding RNA LRRC75A-AS1 facilitates triple negative breast cancer cell proliferation and invasion via functioning as a CeRNA to modulate BAALC. Cell Death Dis. 2020;11(8):643. 10.1038/s41419-020-02821-2. 32811810 10.1038/s41419-020-02821-2 PMC7434919

[2] Shao Zhimin. Interpretation of updates in the Chinese Anti-Cancer association breast cancer guidelines and standards (2024 version) [J]. Chin J Cancer. 2023;33(12). 10.19401/j.cnki.1007-3639.2023.12.004.

[3] Hsieh S-HS 2, Liu S-H. 3, Delayed time from first medical visit to diagnosis for breast cancer patients in Taiwan. 10.1016/j.jfma.2012.12.003 10.1016/j.jfma.2012.12.003 25240303

[4] Patta M, Shankar G, Ahmed F. Why are you late?? A descriptive study of delay in treatment seeking among patients of carcinoma breast presenting to a tertiary care hospital in South India. ICAJ. 2024;0:1–8. 10.25259/icaj_4_2024.

[5] An J, Hershberger PE, Ferrans CE. Delayed presentation, diagnosis, and treatment of breast cancer among Chinese women: an integrative literature review. Cancer Nurs. 2022;46(3):217–32. 10.1097/NCC.0000000000001074. 35283469 10.1097/NCC.0000000000001074

[6] Miller-Kleinhenz JM, Collin LJ, Seidel R, et al. Racial disparities in diagnostic delay among women with breast cancer. J Am Coll Radiol. 2021;18(10):1384–93. 10.1016/j.jacr.2021.06.019. 34280379 10.1016/j.jacr.2021.06.019 PMC8492512

[7] Al’Aref SJ, Anchouche K, Singh G, et al. Clinical applications of machine learning in cardiovascular disease and its relevance to cardiac imaging. Eur Heart J. 2019;40(24):1975–86. 10.1093/eurheartj/ehy404. 30060039 10.1093/eurheartj/ehy404

[8] Fang Y, Zou Y, Xu J, et al. Ambulatory cardiovascular monitoring via a machine-learning-assisted textile triboelectric sensor. Adv Mater. 2021;33(41): 2104178. 10.1002/adma.202104178. 10.1002/adma.202104178 PMC9205313 34467585

[9] Sánchez-Cabo F, Rossello X, Fuster V, et al. Machine learning improves cardiovascular risk definition for young, asymptomatic individuals. J Am Coll Cardiol. 2020;76(14):1674–85. 10.1016/j.jacc.2020.08.017. 33004133 10.1016/j.jacc.2020.08.017

[10] Wang GR, Jiang XL. Investigation on delayed medical treatment of breast cancer patients in Sichuan Province. China J Evid-based Med. 2007;7(10):702–5. 10.3969/j.issn.1672-2531.2007.10.002.

[11] Spitzer RL, Kroenke K, Williams JB, et al. A brief measure for assessing generalized anxiety disorder: the GAD-7. Arch Intern Med. 2006;166(10):1092–7. 10.1001/archinte.166.10.1092. 16717171 10.1001/archinte.166.10.1092

[12] Wang L, Kroenke K, Stump TE, et al. Screening for perinatal depression with the patient health questionnaire depression scale (PHQ-9): A systematic review and meta-analysis. GEN HOSP PSYCHIAT. 2020;68:74–82. 10.1016/j.genhosppsych.2020.12.007. 10.1016/j.genhosppsych.2020.12.007 PMC9112666 33360526

[13] Littlewood K, Cummings DM, Lutes L, et al. Psychometric properties of the family support scale adapted for African American women with type 2 diabetes mellitus. ETHNIC DIS. 2015;25(2):193–9. PMID: 26118148. 26118148

[14] Chen YP, Zhang Y, Chen X, et al. The effects of different surgical approaches on the psychological status, medical coping mode and quality of life of patients with lung cancer. Front Psychol. 2023;14: 1039501. 10.3389/fpsyg.2023.1039501. 37063587 10.3389/fpsyg.2023.1039501 PMC10101174

[15] Pollock SE, Duffy ME. The Health-Related Hardiness Scale: development and psychometric analysis. NURS RES. 1990;39(4):218–22 PMID: 2367202. 2367202

[16] Yarkoni T, Westfall J. Choosing prediction over explanation in psychology: lessons from machine learning. PERSPECT PSYCHOL SCI. 2017;12(6):1100–22. 10.1177/1745691617693393. 28841086 10.1177/1745691617693393 PMC6603289

[17] Alba AC, Agoritsas T, Walsh M, et al. Discrimination and calibration of clinical prediction models: users’ guides to the medical literature. JAMA-J AM MED ASSOC. 2017;318(14):1377–84. 10.1001/jama.2017.12126. 10.1001/jama.2017.12126 29049590

[18] Van Calster B, Wynants L, Verbeek JFM, et al. Reporting and interpreting decision curve analysis: a guide for investigators. Eur Urol. 2018;74(6):796–804. 10.1016/j.eururo.2018.08.038. 30241973 10.1016/j.eururo.2018.08.038 PMC6261531

[19] Lundberg SM, Lee SI. A unified approach to interpreting model predictions. Adv Neural Inf Process Syst. 2017;30.

[20] Dehdar S, Salimifard K, Mohammadi R, et al. Applications of different machine learning approaches in prediction of breast cancer diagnosis delay. Front Oncol. 2023;13: 1103369. 10.3389/fonc.2023.1103369. 36874113 10.3389/fonc.2023.1103369 PMC9978377

[21] Rauscher GH, Ferrans CE, Kaiser K, et al. Misconceptions about breast lumps and delayed medical presentation in urban breast cancer patients. Cancer Epidemiol Biomarkers Prev. 2010;19(3):640–7. 10.1158/1055-9965.EPI-09-0997. 20200436 10.1158/1055-9965.EPI-09-0997 PMC3625394

[22] Nelissen S, Beullens K, Lemal M, et al. Fear of cancer is associated with cancer information seeking, scanning and avoiding: a cross-sectional study among cancer diagnosed and non-diagnosed individuals. Health Inf Libr J. 2015;32(2):107–19. 10.1111/hir.12100. 10.1111/hir.12100 25809822

[23] Alfadul ESA, Tebaig B, Alrawa SS, et al. Delays in presentation, diagnosis, and treatment in Sudanese women with breast cancer: a cross-sectional study. ONCOLOGIST. 2024;29(6):e771–8. 10.1093/oncolo/oyae066. 38642908 10.1093/oncolo/oyae066 PMC11144982

[24] Gullatte MM, Brawley O, Kinney A, et al. Religiosity, spirituality, and cancer fatalism beliefs on delay in breast cancer diagnosis in African American women. J Relig Health. 2009;49(1):62–72. 10.1007/s10943-008-9232-8. 19184437 10.1007/s10943-008-9232-8

[25] Ibrahim NA, Oludara MA. Socio-demographic factors and reasons associated with delay in breast cancer presentation: a study in Nigerian women. Breast. 2012;21(3):416–8. 10.1016/j.breast.2012.02.006. 22381153 10.1016/j.breast.2012.02.006

[26] Gulzar F, Akhtar MS, Sadiq R, et al. Identifying the reasons for delayed presentation of Pakistani breast cancer patients at a tertiary care hospital. Cancer Manag Res. 2019;11:1087–96. 10.2147/CMAR.S180388. 30774437 10.2147/CMAR.S180388 PMC6357878

[27] Ren S, Zhang Y, Qin P, et al. Factors influencing total delay of breast cancer in northeast of China. Front Oncol. 2022;12: 841438. 10.3389/fonc.2022.841438. 35311134 10.3389/fonc.2022.841438 PMC8924654

[28] Ma J, Luo Y, Yang S, et al. Patient delay and related influencing factors in Chinese women under 35 years diagnosed with cervical cancer: A cross-sectional study. ASIA-PAC J ONCOL NUR. 2022;10(2):100165. 10.1016/j.apjon.2022.100165. 10.1016/j.apjon.2022.100165 PMC9791130 36579173

