# Supplementary material for: Development and validation of a machine learning model to predict delays in seeking medical care among patients with breast cancer in China
Source: BMC Cancer. 2025 Sep 30;25:1442. doi: 10.1186/s12885-025-14813-6 (PMC12482459; doi:10.1186/s12885-025-14813-6)
Supplement: Supplementary file 1 — Supplementary Material 1. [file 12885_2025_14813_MOESM1_ESM.docx]

**Table 1 Demographic and clinical characteristics of the participants**

| **Variables** | | **Non-delay** (n=328) | **Delay** (n=212) | **Total(=540** | **S**tatistic | | | ***P*** |
| --- | --- | --- | --- | --- | --- | --- | --- | --- |
| Place of residence, n (%) | | | | | | | | |
|  | Rural | 146(44.5%) | 109(51.4%) | 255(47.22%) | | 2.46 | 0.117 | |
|  | Urban | 182(55.5%) | 103(48.6%) | 285(52.78%) | |  |  | |
| House hold situation, n (%) | | | | | | | | |
|  | Alone | 12(3.7%) | 13(6.1%) | 25(4.63%) | | 1.78 | 0.182 | |
|  | Social | 316(96.3%) | 199(93.9%) | 515(95.37%) | |  |  | |
| Number of family members, n (%) | | | | | | | | |
|  | One-three | 136(41.5%) | 91(42.9%) | 227(42.04%) | | 0.42 | 0.812 | |
|  | Four-five | 145(44.2%) | 88(41.5%) | 233(43.15%) | |  |  | |
|  | Six and above | 47(14.3%) | 33(15.6%) | 80(14.81%) | |  |  | |
| Ethnicity, n (%) | | | | | | | | |
|  | Han Chinese | 324(98.8%) | 203(95.8%) | 527(97.59%) | | 5.02 | 0.025 | |
|  | Others ethnicity | 4(1.2%) | 9(4.2%) | 13(2.41%) | |  |  | |
| Religion, n (%) | | | | | | | | |
|  | Yes | 3(0.9%) | 13(6.1%) | 16(2.96%) | | 12.19 | <0.001 | |
|  | No | 325(99.1%) | 199(93.9%) | 524(97.04%) | |  |  | |
| Education levels, n (%) | | | | | | | | |
|  | Illiterate | 19(5.8%) | 26(12.3%) | 45(8.33%) | | 15.66 | 0.008 | |
|  | Primary school | 76(23.2%) | 53(25.1%) | 129(23.89%) | |  |  | |
|  | Middle school | 100(30.5%) | 77(36.3%) | 177(32.78%) | |  |  | |
|  | High school | 54(16.5%) | 23(10.8%) | 77(14.26%) | |  |  | |
|  | Junior college | 43(13.1%) | 20(9.4%) | 63(11.67%) | |  |  | |
|  | University or above | 36(10.9%) | 13(6.1%) | 49(9.07%) | |  |  | |
| Employment, n (%) | | | | | | | | |
|  | Farmer | 139(42.4%) | 101(47.6%) | 240(44.44%) | | 11.7 | 0.111 | |
|  | Worker | 22(6.7%) | 20(9.4%) | 42(7.78%) | |  |  | |
|  | Businessmen | 13(4%) | 6(2.8%) | 19(3.52%) | |  |  | |
|  | Service industry | 33(10%) | 16(7.5%) | 49(9.07%) | |  |  | |
|  | Management industry | 17(5.2%) | 6(2.8%) | 23(4.26%) | |  |  | |
|  | Technical industry | 16(4.9%) | 2(0.9%) | 18(3.33%) | |  |  | |
|  | Retirement | 41(12.5%) | 25(11.8%) | 66(12.22%) | |  |  | |
|  | Others | 47(14.3%) | 36(16.9%) | 83(15.37%) | |  |  | |
| Marital status, n (%) | | | | | | | | |
|  | Married | 294(89.6%) | 184(86.8%) | 478(88.52%) | | 2.51 | 0.473 | |
|  | Unmarried | 6(1.8%) | 6(2.8%) | 12(2.22%) | |  |  | |
|  | Divorced | 16(4.9%) | 9(4.2%) | 25(4.63%) | |  |  | |
|  | Widowed | 12(3.7%) | 13(6.2%) | 25(4.63%) | |  |  | |
| Monthly income (RMB), n (%) | | | | | | | | |
|  | Below 3000 | 145(44.2%) | 117(55.2%) | 262(48.52%) | | 9.23 | 0.026 | |
|  | 3000-5000 | 123(37.5%) | 70(33%) | 193(35.74%) | |  |  | |
|  | 5000-8000 | 33(10.1%) | 18(8.5%) | 51(9.44%) | |  |  | |
|  | More than 8000 | 27(8.2%) | 7(3.3%) | 34(6.3%) | |  |  | |
| Smoking status, n (%) | | | | | | | | |
|  | Never | 308(94%) | 198(93.4%) | 506(93.7%) | | 3.47 | 0.185 | |
|  | Occasionally | 8(2.4%) | 10(4.7%) | 18(3.33%) | |  |  | |
|  | Often | 12(3.6%) | 4(1.9%) | 16(2.96%) | |  |  | |
| Alcohol status, n (%) | | | | | | | | |
|  | Never | 245(74.7%) | 167(78.8%) | 412(76.3%) | | 3.12 | 0.21 | |
|  | Occasionally | 74(22.6%) | 36(17%) | 110(20.37%) | |  |  | |
|  | Often | 9(2.7%) | 9(4.2%) | 18(3.33%) | |  |  | |
| Medical payment method, n (%) | | | | | | | | |
|  | Self-payment | 19(5.8%) | 11(5.2%) | 30(5.56%) | | 9.74 | 0.008 | |
|  | Employee medical insurance | 116(35.4%) | 49(23.1%) | 165(30.56%) | |  |  | |
|  | New rural health insurance | 193(58.8%) | 152(71.7%) | 345(63.89%) | |  |  | |
| Comorbidities, n (%) | | | | | | | | |
|  | No | 254(77,4%) | 161(75.9%) | 415(76.85%) | | 0.16 | 0.687 | |
|  | Yes | 74(22.6%) | 51(24.1%) | 125(23.15%) | |  |  | |
| Conscious severity of breast disease, n (%) | | | | | | | | |
|  | Not serious | 48(14.6%) | 30(14.1%) | 78(14.44%) | | 2.32 | 0.509 | |
|  | Moderate | 111(33.8%) | 60(28.3%) | 171(31.67%) | |  |  | |
|  | Rather serious | 127(38,7%) | 89(42%) | 216(40%) | |  |  | |
|  | Very serious | 42(12.9%) | 33(15.6%) | 75(13.89%) | |  |  | |
| Status of understanding breast diseases, n (%) | | | | | | | | |
|  | Understand | 99(30.2%) | 77(36.3%) | 176(32.59%) | | 2.21 | 0.137 | |
|  | Don't understand | 229(69.8%) | 135(63.7%) | 364(67.41%) | |  |  | |
| Physical examination status, n (%) | | | | | | | | |
|  | Once a year | 90(27.4%) | 48(22.6%) | 138(25.56%) | | 37.79 | <0.001 | |
|  | Every two years | 34(10.4%) | 10(4.7%) | 44(8.15%) | |  |  | |
|  | Three years or above | 18(54.9%) | 3(1.4%) | 21(3.89%) | |  |  | |
|  | Occasionally | 103(31.4%) | 46(21.7%) | 149(27.59%) | |  |  | |
|  | Never | 83(25.3%) | 105(49.5%) | 188(34.81%) | |  |  | |
| Healthcare workers, n (%) | | | | | | | | |
|  | No | 265(80.8%) | 188(88.7%) | 453(83.89%) | | 5.93 | 0.015 | |
|  | Yes | 63(19.2%) | 24(11.3%) | 87(16.11%) | |  |  | |
| Medical choice, n (%) | | | | | | | | |
|  | First level hospital | 228(69.5%) | 69(32.5%) | 297(55%) | | 77.45 | <0.001 | |
|  | Second level hospital | 32(9.8%) | 26(12.3%) | 58(10.74%) | |  |  | |
|  | Third level hospital | 63(19.2%) | 107(50.5%) | 170(31.48%) | |  |  | |
|  | The small clinic | 5(1.5%) | 10(4.7%) | 15(2.78%) | |  |  | |
| Barriers and reasons for the delay in seeking medical care, n (%) | | | | | | | | |
|  | Economic hardship | 200(61%) | 101(47.6%) | 301(55.74%) | | 21.44 | 0.002 | |
|  | Mild symptoms of self-awareness | 69(21%) | 75(35.4%) | 144(26.67%) | |  |  | |
|  | Unaccompanied | 27(8.2%) | 9(4.2%) | 36(6.67%) | |  |  | |
|  | Too far away | 18(5%) | 11(5.2%) | 29(5.37%) | |  |  | |
|  | Untreatable | 3(0.9%) | 1(0.5%) | 4(0.74%) | |  |  | |
|  | Taboo/avoidance | 9(3%) | 11(5.2%) | 20(3.7%) | |  |  | |
|  | No time available | 2(0.9%) | 4(1.9%) | 6(1.11%) | |  |  | |
| The preferred solution for feeling breast discomfort, n (%) | | | | | | | | |
|  | Not seeking medical treatment | 8(2.4%) | 30(14.2%) | 38(7.04%) | | 41.7 | <0.001 | |
|  | Taking medicine | 45(14%) | 51(24.1%) | 96(17.78%) | |  |  | |
|  | Seeking medical facility | 273(83%) | 129(60.8%) | 402(74.44%) | |  |  | |
|  | Online consultation | 2(0.6%) | 2(0.9%) | 4(0.74%) | |  |  | |
| Channels for acquiring health knowledge, n (%) | | | | | | | | |
|  | Not interested | 50(15.2%) | 52(24.5%) | 102(18.89%) | | 12.49 | 0.052 | |
|  | TV | 70(21.3%) | 36(17.1%) | 106(19.63%) | |  |  | |
|  | Magazine | 14(4.3%) | 5(2.3%) | 19(3.52%) | |  |  | |
|  | Online | 95(29.1%) | 58(27.3%) | 153(28.33%) | |  |  | |
|  | Health lecture | 9(2.7%) | 2(0.9%) | 11(2.04%) | |  |  | |
|  | Talking with family | 87(26.5%) | 54(25.5%) | 141(26.11%) | |  |  | |
|  | Training course | 3(0.9%) | 5(2.4%) | 8(1.48%) | |  |  | |
| First detected symptoms, n (%) | | | | | | | | |
|  | Breast lump | 291(88.7%) | 189(89.2%) | 480(88.89%) | | 5.8 | 0.214 | |
|  | Nipple ulcers/itching | 3(1%) | 4(1.9%) | 7(1.3%) | |  |  | |
|  | Change in breast shape | 4(1.2%) | 7(3.3%) | 11(2.04%) | |  |  | |
|  | Nipple discharge4 | 6(1.8%) | 3(1.4%) | 9(1.67%) | |  |  | |
|  | No symptom (no examination) | 24(7.3%) | 9(4.2%) | 33(6.11%) | |  |  | |
| Method of symptom discovery, n (%) | | | | | | | | |
|  | Pain stimulation | 52(15.8%) | 45(21.2%) | 97(17.96%) | | 2.79 | 0.425 | |
|  | Accidental discovery | 165(50.3%) | 103(48.6%) | 268(49.63%) | |  |  | |
|  | Breast self-examination | 56(17.1%) | 34(16%) | 90(16.67%) | |  |  | |
|  | Physical/clinical breast examination | 55(16.8%) | 30(14.2%) | 85(15.74%) | |  |  | |
| Companion, n (%) | | | | | | | | |
|  | Spouse | 214(65.2%) | 135(63.7%) | 349(64.63%) | | 2.35 | 0.309 | |
|  | Family | 88(26.8%) | 52(24.5%) | 140(25.93%) | |  |  | |
|  | Friends | 26(8%) | 25(11.8%) | 51(9.44%) | |  |  | |
| Hospitals with confirmed breast diseases, n (%) | | | | | | | | |
|  | Third level hospital | 217(66.1%) | 144(68%) | 361(66.85%) | | 0.73 | 0.693 | |
|  | Second level hospital | 57(17.4%) | 31(14.6%) | 88(16.3%) | |  |  | |
|  | First level hospital | 54(16.5%) | 37(17.4%) | 91(16.85%) | |  |  | |
| Pathologic stage, n (%) | | | | | | | | |
|  | I | 103(31.4%) | 66(31.1%) | 169(31.3%) | | 6.07 | 0.108 | |
|  | II | 126(38.4%) | 63(29.7%) | 189(35%) | |  |  | |
|  | III | 51(15.5%) | 40(18.9%) | 91(16.85%) | |  |  | |
|  | IV | 48(14.7%) | 43(20.3%) | 91(16.85%) | |  |  | |
| Family history of breast cancer, n (%) | | | | | | | | |
|  | yes | 18(5.5%) | 11(5.2%) | 29(5.37%) | | 0.02 | 0.88 | |
|  | No | 310(94.5%) | 201(94.8%) | 511(94.63%) | |  |  | |
| Family history of other cancer, n (%) | | | | | | | | |
|  | Yes | 23(7.1%) | 24(11.3%) | 47(8.7%) | | 3.01 | 0.083 | |
|  | No | 305(92.9%) | 188(88.7%) | 493(91.3%) | |  |  | |
| Age at diagnosis years, n (%) | | | | | | | | |
|  | 18-44 | 83(25.3%) | 39(18.4%) | 122(22.59%) | | 3.57 | 0.167 | |
|  | 45-59 | 195(59.5%) | 136(64.2%) | 331(61.3%) | |  |  | |
|  | ≥60 | 50(15.2%) | 37(17.4%) | 87(16.11%) | |  |  | |
| Distance from the hospital, median [IQR] | | 9.00[4.00,18.00] | 41.00[20.00,50.00] |  | | -11.55 | <0.001 | |
| Anxiety, median [IQR] | | 4.00[0.00,7.00] | 4.00[0.00,7.00] |  | | -0.34 | 0.731 | |
| Depression, median [IQR] | | 4.00[1.00,7.00] | 4.00[1.00,8.00] |  | | -0.45 | 0.651 | |
| Family support, median [IQR] | | 34.00[32.00,36.00] | 34.00[33.00,36.00] |  | | -0.39 | 0.692 | |
| **Medical coping mode** | | | | | | | | |
| Confrontation, median [IQR] | | 21.00[19.00,22.00] | 21.00[19.00,22.00] |  | | 0.78 | 0.43 | |
| Avoidance, median [IQR] | | 17.00[15.00,19.00] | 17.00[15.00,18.00] |  | | 1.52 | 0.126 | |
| Acceptance resignation, median [IQR] | | 14.00[13.00,15.00] | 14.00[13.00,15.00] |  | | 0.63 | 0.52 | |
| **Health hardiness** | | | | | | | | |
| Health value, median [IQR] | | 23.00[21.00,24.00] | 23.00[19.00,24.00] |  | | 2.29 | 0.02 | |
| Internal health locus of control, median [IQR] | | 18.00[15.00,20.00] | 17.00[15.00,19.00] |  | | 1.81 | 0.069 | |
| External health locus of control, median [IQR] | | 20.00[17.00,23.00] | 20.00[17.00,23.00] |  | | -0.19 | 0.847 | |
| Perceived health competence, median [IQR] | | 20.00[17.00,22.00] | 19.00[17.00,21.00] |  | | 1.04 | 0.296 | |
