# Supplementary material for: Development and validation of a machine learning model to predict delays in seeking medical care among patients with breast cancer in China
Source: BMC Cancer. 2025 Sep 30;25:1442. doi: 10.1186/s12885-025-14813-6 (PMC12482459; doi:10.1186/s12885-025-14813-6)
Supplement: Supplementary file 2 — Supplementary Material 2. [file 12885_2025_14813_MOESM2_ESM.docx]

**Table 2. The comparison of demographic and clinical characteristics between training and verification set.**

| **Variables** | |  | **Training (n=378)** | | **Verification(n=162)** | | | **Statistic** | | **P** |
| --- | --- | --- | --- | --- | --- | --- | --- | --- | --- | --- |
| Place of residence, n(%) | | | | | | | | | | |
|  | Rural | | | 182(48.15) | | 73(45.06) | 0.43 | | 0.510 | |
|  | Urban | | | 196(51.85) | | 89(54.94) |  | |  | |
| House hold situation, n(%) | | | | | | | | | | |
|  | Alone | | | 16(4.23) | | 9(5.56) | 0.45 | | 0.503 | |
|  | Social | | | 362(95.77) | | 153(94.44) |  | |  | |
| Number of family members, n(%) | | | | | | | | | | |
|  | One-three | | | 162(42.86) | | 65(40.12) | 0.74 | | 0.692 | |
|  | Four-five | | | 163(43.12) | | 70(43.21) |  | |  | |
|  | Six and above | | | 53(14.02) | | 27(16.67) |  | |  | |
| Ethnicity, n(%) | | | | | | | | | | |
|  | Han Chinese | | | 162(42.86) | | 65(40.12) | 0.74 | | 0.692 | |
|  | Others ethnicity | | | 163(43.12) | | 70(43.21) |  | |  | |
| Religion, n(%) | | | | | | | | | | |
|  | Yes | | | 14(3.70) | | 2(1.23) | 2.40 | | 0.121 | |
|  | No | | | 364(96.30) | | 160(98.77) |  | |  | |
| Education levels, n(%) | | | | | | | | | | |
|  | Illiterate | | | 30(7.94) | | 15(9.26) | 6.32 | | 0.276 | |
|  | Primary school | | | 100(26.46) | | 29(17.90) |  | |  | |
|  | Middle school | | | 117(30.95) | | 60(37.04) |  | |  | |
|  | High school | | | 54(14.29) | | 23(14.20) |  | |  | |
|  | Junior college | | | 46(12.17) | | 17(10.49) |  | |  | |
|  | University or above | | | 31(8.20) | | 18(11.11) |  | |  | |
| Employment, n(%) | | | | | | | | | | |
|  | Farmer | | | 170(44.97) | | 70(43.21) | 3.38 | | 0.848 | |
|  | Worker | | | 31(8.20) | | 11(6.79) |  | |  | |
|  | Businessmen | | | 13(3.44) | | 6(3.70) |  | |  | |
|  | Service industry | | | 36(9.52) | | 13(8.02) |  | |  | |
|  | Management industry | | | 14(3.70) | | 9(5.56) |  | |  | |
|  | Technicial industry | | | 10(2.65) | | 8(4.94) |  | |  | |
|  | Retirement | | | 46(12.17) | | 20(12.35) |  | |  | |
|  | Others | | | 58(15.34) | | 25(15.43) |  | |  | |
| Marital status, n(%) | | | | | | | | | | |
|  | Married | | | 336(88.89) | | 142(87.65) | 2.78 | | 0.427 | |
|  | Unmarried | | | 6(1.59) | | 6(3.70) |  | |  | |
|  | Divorced | | | 17(4.50) | | 8(4.94) |  | |  | |
|  | Widowed | | | 19(5.03) | | 6(3.70) |  | |  | |
| Monthly income RMB, n(%) | | | | | | | | | | |
|  | Below 3000 | | | 145(44.21) | | 117(55.19) | 9.23 | | 0.026 | |
|  | 3000-5000 | | | 123(37.50) | | 70(33.02) |  | |  | |
|  | 5000-8000 | | | 33(10.06) | | 18(8.49) |  | |  | |
|  | More than 8000 | | | 27(8.23) | | 7(3.30) |  | |  | |
| Smoking status, n(%) | | | | | | | | | | |
|  | Never | | | 351(92.86) | | 155(95.68) | 3.17 | | 0.205 | |
|  | Occasionally | | | 16(4.23) | | 2(1.23) |  | |  | |
|  | Often | | | 11(2.91) | | 5(3.09) |  | |  | |
| Alcohol status, n(%) | | | | | | | | | | |
|  | Never | | | 287(75.93) | | 125(77.16) | 0.11 | | 0.947 | |
|  | Occasionally | | | 78(20.63) | | 32(19.75) |  | |  | |
|  | Often | | | 13(3.44) | | 5(3.09) |  | |  | |
| Medical payment method, n(%) | | | | | | | | | | |
|  | Self-payment | | | 17(4.50) | | 13(8.02) | 2.90 | | 0.235 | |
|  | Employee medical insurance | | | 119(31.48) | | 46(28.40) |  | |  | |
|  | New rural health insurance | | | 242(64.02) | | 103(63.58) |  | |  | |
| Comorbidities, n(%) | | | | | | | | | | |
|  | No | | | 285(75.40) | | 130(80.25) | 1.50 | | 0.221 | |
|  | Yes | | | 93(24.60) | | 32(19.75) |  | |  | |
| Conscious severity of breast disease, n(%) | | | | | | | | | | |
|  | Not serious | | | 55(14.55) | | 23(14.20) | 0.63 | | 0.888 | |
|  | Moderate | | | 120(31.75) | | 51(31.48) |  | |  | |
|  | Rather serious | | | 148(39.15) | | 68(41.98) |  | |  | |
|  | Very serious | | | 55(14.55) | | 20(12.35) |  | |  | |
| Status of understanding breast diseases, n(%) | | | | | | | | | | |
|  | Understand | | | 128(33.86) | | 48(29.63) | 0.92 | | 0.336 | |
|  | Don't understand | | | 250(66.14) | | 114(70.37) |  | |  | |
| Physical examination status, n(%) | | | | | | | | | | |
|  | Once a year | | | 95(25.13) | | 43(26.54) | 4.75 | | 0.314 | |
|  | Every two years | | | 33(8.73) | | 11(6.79) |  | |  | |
|  | Three years or above | | | 17(4.50) | | 4(2.47) |  | |  | |
|  | Occasionally | | | 96(25.40) | | 53(32.72) |  | |  | |
|  | Never | | | 137(36.24) | | 51(31.48) |  | |  | |
| Healthcare workers, n(%) | | | | | | | | | | |
|  | No | | | 312(82.54) | | 141(87.04) | 1.70 | | 0.193 | |
|  | Yes | | | 66(17.46) | | 21(12.96) |  | |  | |
| Medical choice, n(%) | | | | | | | | | | |
|  | First level hospital | | | 200(52.91) | | 97(59.88) | 3.59 | | 0.309 | |
|  | Second level hospital | | | 42(11.11) | | 16(9.88) |  | |  | |
|  | Third level hospital | | | 123(32.54) | | 47(29.01) |  | |  | |
|  | The small clinic | | | 13(3.44) | | 2(1.23) |  | |  | |
| Barriers and reasons for the delay in seeking a medical care, n(%) | | | | | | | | | | |
|  | Economic hardship | | | 207(54.76) | | 94(58.02) | 2.85 | | 0.827 | |
|  | Mild symptoms of self-awareness | | | 102(26.98) | | 42(25.93) |  | |  | |
|  | Unaccompanied | | | 27(7.14) | | 9(5.56) |  | |  | |
|  | Too far away | | | 20(5.29) | | 9(5.56) |  | |  | |
|  | Untreatable | | | 3(0.79) | | 1(0.62) |  | |  | |
|  | Taboo/avoidance | | | 16(4.23) | | 4(2.47) |  | |  | |
|  | No time available | | | 3(0.79) | | 3(1.85) |  | |  | |
| The preferred solution for feeling breast discomfort, n(%) | | | | | | | | | | |
|  | Not seeking medical treatment | | | 29(7.67) | | 9(5.56) | 1.78 | | 0.619 | |
|  | Taking medicine | | | 69(18.25) | | 27(16.67) |  | |  | |
|  | Seeking medical facility | | | 278(73.54) | | 124(76.54) |  | |  | |
|  | Online consultation | | | 2(0.53) | | 2(1.23) |  | |  | |
| Channels for acquiring health knowledge, n(%) | | | | | | | | | | |
|  | Not interested | | | 82(21.69) | | 20(12.35) | 9.90 | | 0.129 | |
|  | TV | | | 78(20.63) | | 28(17.28) |  | |  | |
|  | Magazine | | | 14(3.70) | | 5(3.09) |  | |  | |
|  | Online | | | 100(26.46) | | 53(32.72) |  | |  | |
|  | Health lecture | | | 7(1.85) | | 4(2.47) |  | |  | |
|  | Talking with family | | | 91(24.07) | | 50(30.86) |  | |  | |
|  | Training course | | | 6(1.59) | | 2(1.23) |  | |  | |
| First detected symptoms, n(%) | | | | | | | | | | |
|  | Breast lump | | | 336(88.89) | | 144(88.89) | 7.41 | | 0.116 | |
|  | Nipple ulcers/itching | | | 2(0.53) | | 5(3.09) |  | |  | |
|  | Change in breast shape | | | 8(2.12) | | 3(1.85) |  | |  | |
|  | Nipple discharge4 | | | 8(2.12) | | 1(0.62) |  | |  | |
|  | No symptom (no examination) | | | 24(6.35) | | 9(5.56) |  | |  | |
| Method of symptom discovery, n(%) | | | | | | | | | | |
|  | Pain stimulation | | | 71(18.78) | | 26(16.05) | 1.77 | | 0.622 | |
|  | Accidental discovery | | | 182(48.15) | | 86(53.09) |  | |  | |
|  | Breast self-examination | | | 62(16.40) | | 28(17.28) |  | |  | |
|  | Physical/clinical breast examination | | | 63(16.67) | | 22(13.58) |  | |  | |
| Companion, n(%) | | | | | | | | | | |
|  | Spouse | | | 239(63.23) | | 110(67.90) | 1.24 | | 0.537 | |
|  | Family | | | 103(27.25) | | 37(22.84) |  | |  | |
|  | Friends | | | 36(9.52) | | 15(9.26) |  | |  | |
| Hospitals with confirmed breast diseases, n(%) | | | | | | | | | | |
|  | Third level hospital | | | 246(65.08) | | 115(70.99) | 3.40 | | 0.183 | |
|  | Second level hospital | | | 61(16.14) | | 27(16.67) |  | |  | |
|  | First level hospital | | | 71(18.78) | | 20(12.35) |  | |  | |
| Pathologic stage, n(%) | | | | | | | | | | |
|  | I | | | 130(34.39) | | 39(24.07) | 5.90 | | 0.117 | |
|  | II | | | 124(32.80) | | 65(40.12) |  | |  | |
|  | III | | | 62(16.40) | | 29(17.90) |  | |  | |
|  | IV | | | 62(16.40) | | 29(17.90) |  | |  | |
| Family history of breast cancer, n(%) | | | | | | | | | | |
|  | yes | | | 19(5.03) | | 10(6.17) | 0.29 | | 0.588 | |
|  | No | | | 359(94.97) | | 152(93.83) |  | |  | |
| Family history of others cancer, n(%) | | | | | | | | | | |
|  | Yes | | | 32(8.47) | | 15(9.26) | 0.09 | | 0.764 | |
|  | No | | | 346(91.53) | | 147(90.74) |  | |  | |
| Age at diagnosed years, n(%) | | | | | | | | | | |
|  | 18-44 | | | 84(22.22) | | 38(23.46) | 0.64 | | 0.725 | |
|  | 45-59 | | | 230(60.85) | | 101(62.35) |  | |  | |
|  | ≥60 | | | 64(16.93) | | 23(14.20) |  | |  | |
| Distance from the hospital, median[IQR] | | | | 14.00[5.00,41.00] | | 16.00[5.00,40.00] | -0.34 | | 0.733 | |
| Anxiety, median[IQR] | | | | 4.00[0.00,7.00] | | 4.00[0.00,7.00] | 0.42 | | 0.673 | |
| Depression, median[IQR] | | | | 4.00[1.00,8.00] | | 4.00[1.00,7.00] | 0.50 | | 0.617 | |
| Family support, median[IQR] | | | | 34.00[33.00,36.00] | | 34.00[32.00,36.00] | 1.00 | | 0.314 | |
| **Medical coping mode** | | | | | | | | | | |
| Confrontation, median[IQR] | | | | 21.00[19.00,22.00] | | 21.00[20.00,22.00] | 0.54 | | 0.586 | |
| Avoidance, median[IQR] | | | | 17.00[15.00,18.00] | | 17.00[15.00,19.00] | -1.71 | | 0.085 | |
| Acceptance resignation, median[IQR] | | | | 14.00[13.00,15.00] | | 14.00[13.00,15.00] | -0.21 | | 0.833 | |
| **Health hardiness** | | | | | | | | | | |
| Health value, median[IQR] | | | | 23.00[20.00,24.00] | | 23.00[20.00,24.00] | 0.45 | | 0.646 | |
| Internal health locus of control, median[IQR] | | | | 17.00[15.00,20.00] | | 17.00[15.00,19.00] | 1.50 | | 0.131 | |
| External health locus of control, median[IQR] | | | | 20.00[17.00,24.00] | | 20.00[16.00,23.00] | 1.59 | | 0.110 | |
| Perceived health competence, median[IQR] | | | | 19.00[17.00,22.00] | | 19.00[17.00,21.00] | -0.10 | | 0.923 | |
